# Correction: Porto et al. The Effects of Frost and Fire on the Traits, Resources, and Floral Visitors of a Cerrado Plant, and Their Impact on the Plant–Visitor Interaction Network and Fruit Formation. *Plants* 2025, *14*, 1977

**DOI:** 10.3390/plants14193027

**Published:** 2025-09-30

**Authors:** Gabriela Fraga Porto, José Henrique Pezzonia, Ludimila Juliele Carvalho Leite, Jordanny Luiza Sousa Santos, Kleber Del-Claro

**Affiliations:** 1 Programa de Pós-Graduação em Entomologia, Faculdade de Filosofia, Ciências e Letras de Ribeirão Preto—FFCLRP, Universidade de São Paulo—USP, Ribeirão Preto 14040-901, Brazil; gabrielafraga@usp.br (G.F.P.); jh.pezzonia@usp.br (J.H.P.); ludimila.leite@usp.br (L.J.C.L.); 2 Instituto de Biologia, Universidade Federal de Uberlândia, Uberlândia 38400-902, Brazil; jordanny.santos@ufu.br

An error occurred in the original publication [1] regarding the formatting of the references. During the process of adapting the citations to the journal’s style, an artificial intelligence tool was used which, in addition to reformatting the references, introduced unintended modifications, including replacement of journal titles, alteration of volume/issue numbers [5,15,18,20,23,26,27,61] and, in some cases, the generation of non-existent references [12,17,24]. These inconsistencies were not detected at the time of submission.

During the correction process, the reference list was carefully re-checked, all inaccuracies were corrected, and the corresponding DOIs were added where available to ensure completeness and accuracy [6,7,35,64,66]. The revised reference list has been updated in the manuscript, and all corrected references are listed here.

5. Lazarina, M.; Sgardelis, S.P.; Tscheulin, T.; Kallimanis, A.S.; Devalez, J.; Petanidou, T. Bee Response to Fire Regimes in Mediterranean Pine Forests: The Role of Nesting Preference, Trophic Specialization, and Body Size. *Basic Appl. Ecol.* **2016**, *17*, 308–320. https://doi.org/10.1016/j.baae.2016.02.001.
6. Antonio, A.C.; Scalon, M.C.M.; Rossatto, D.R. The Role of Bud Protection and Bark Density in Frost Resistance of Savanna Trees. *Plant Biol.* **2019**, *22*, 55–61. https://doi.org/10.1111/plb.13050.
7. Hoffmann, W.A.; Flake, S.W.; Abreu, R.C.; Pilon, N.A.; Rossatto, D.R.; Durigan, G. Rare frost events reinforce tropical savanna–forest boundaries. *J. Ecol.* **2019**, *107*, 468–477. https://doi.org/10.1111/1365-2745.13047.
12. Morton, E.M.; Rafferty, N.E. Plant–Pollinator Interactions under Climate Change: The Use of Spatial and Temporal Transplants. *Appl. Plant Sci.* **2017**, *5*, 1600133. https://doi.org/10.3732/apps.1600133.
15. Morente-López, J.; Lara-Romero, C.; Ornosa, C.; Iriondo, J.M. Phenology Drives Species Interactions and Modularity in a Plant—Flower Visitor Network. *Sci. Rep*. **2018**, *8*, 9386. https://doi.org/10.1038/s41598-018-27725-2.
17. Layek, U.; Das, U.; Karmakar, P. The Pollination Efficiency of a Pollinator Depends on Its Foraging Strategy, Flowering Phenology, and the Flower Characteristics of a Plant Species. *J. Asia-Pac. Entomol.* **2022**, *25*, 101882. https://doi.org/10.1016/j.aspen.2022.101882.
18. Peralta, G.; CaraDonna, P.J.; Rakosy, D.; Fründ, J.; Tudanca, M.P.P.; Dormann, C.F.; Burkle, L.A.; Kaiser-Bunbury, C.N.; Knight, T.M.; Resasco, J.; et al. Predicting Plant–Pollinator Interactions: Concepts, Methods, and Challenges. *Trends Ecol. Evol.* **2024**, *39*, 494–505. https://doi.org/10.1016/j.tree.2023.12.005.
20. Welti, E.A.R.; Joern, A. Fire and Grazing Modulate the Structure and Resistance of Plant–Floral Visitor Networks in a Tallgrass Prairie. *Oecologia* **2018**, *186*, 517–528. https://doi.org/10.1007/s00442-017-4019-9.
23. Zografou, K.; Swartz, M.T.; Tilden, V.P.; McKinney, E.N.; Eckenrode, J.A.; Sewall, B.J. Stable Generalist Species Anchor a Dynamic Pollination Network. *Bull. Ecol. Soc. Am.* **2020**, *101*, e01779. https://doi.org/10.1002/bes2.1779.
24. Clavel, J.; Julliard, R.; Devictor, V. Worldwide Decline of Specialist Species: Toward a Global Functional Homogenization? *Front. Ecol. Environ.* **2011**, *9*, 222–228. https://doi.org/10.1890/080216.
26. de Deus, F.F.; Oliveira, P.E. Changes in Floristic Composition and Pollination Systems in a “Cerrado” Community after 20 Years of Fire Suppression. *Braz. J. Bot.* **2016**, *39*, 1051–1063. https://doi.org/10.1007/s40415-016-0304-9.
27. Aizen, M.A.; Sabatino, M.; Tylianakis, J.M. Specialization and Rarity Predict Nonrandom Loss of Interactions from Mutualist Networks. *Science* **2012**, *335*, 1486–1489. https://doi.org/10.1126/science.1215320.
35. Maruyama, P.K.; Bosenbecker, C.; Cardoso, J.C.F.; Sonne, J.; Ballarin, C.S.; Souza, C.S.; Leguizamón, J.; Lopes, A.V.; Maglianesi, M.A.; Otárola, M.F.; et al. Urban Environments Increase Generalization of Hummingbird–Plant Networks across Climate Gradients. *Proc. Natl. Acad. Sci. USA* **2024**, *121*, e2322347121. https://doi.org/10.1073/pnas.2322347121.
61. de Antonio, A.C.; Scalon, M.C.; Rossatto, D.R. Leaf Size and Thickness Are Related to Frost Damage in Ground Layer Species of Neotropical Savannas. *Flora* **2023**, *299*, 152208. https://doi.org/10.1016/j.flora.2022.152208.
64. Whitecross, M.A.; Archibald, S.A.; Witkowski, E.T.F. Do Freeze Events Create a Demographic Bottleneck for *Colophospermum mopane*? *S. Afr. J. Bot.* **2012**, *83*, 9–18. https://doi.org/10.1016/j.sajb.2012.07.008.
66. e Silva, S.C.O.; Souza, C.S.; de Araújo, W.S. Individual-based plant-visitor networks in Brazilian palm swamps under different dryness levels. *Plant Ecol.* **2024**, *225*, 543–554. https://doi.org/10.1007/s11258-024-01410-z.

The authors state that the scientific conclusions are unaffected. This correction was approved by the Academic Editor. The original publication has also been updated.
